# Health sciences library leadership skills in an interprofessional landscape: a review and textual analysis

**DOI:** 10.5195/jmla.2020.917

**Published:** 2020-10-01

**Authors:** Nicole Capdarest-Arest, Jamie M. Gray

**Affiliations:** 1 ncapdarest@ucdavis.edu, Blaisdell Medical Library, University of California, Davis, CA; 2 jmg2015@qatar-med.cornell.edu, Distributed eLibrary, Weill Cornell Medicine–Qatar, Doha, Qatar

## Abstract

Academic medical libraries sit at the crossroads of the complex landscape of the health sciences. Medical librarians in these environments must navigate and lead endeavors and services that involve many professions. In addition to being excellent leaders in their own professions, medical librarians must also improve their skills in leading in an interprofessional context by informing themselves of the qualities and skills valued in connected professions. In this project, the authors set out to understand leadership principles from three professions closely affiliated with medical librarianship to identify a core interdisciplinary leadership skill set. To do so, we conducted a mapping review of the existing literature from the last five years around leadership in academic medicine, academic nursing, hospital administration, and medical librarianship to identify core leadership skills across the disciplines and discover potential differences. We used text analysis and descriptive analysis to extract skills that were mentioned and uncover trends in the identified literature. Modern medical librarians must extend their leadership beyond the internal library setting, particularly as they become more involved with connecting and collaborating with leaders across disciplines. To successfully navigate such an interdisciplinary landscape and enhance the impact of the library in the broader organization, it is important to have the skills and vocabulary of leadership across the various professions.

## INTRODUCTION

Academic health sciences libraries sit at the crossroads of the complex landscape of the health sciences. Health sciences librarians in these environments must navigate and lead endeavors and provide services that involve many professions. In one day, for example, a health sciences library leader could meet with a head of the medicine department, a director of nursing education, and a chief information officer, as well as a patient safety director and an assistant dean of medical education, each of whom are held to the leadership standards of their respective fields. In addition to being excellent leaders in their own professions, how can health sciences librarians lead in an interprofessional context? In this project, the authors set out to understand leadership principles from three professions that are closely affiliated with health sciences librarianship to begin identifying a core interdisciplinary leadership skill set.

### Leadership theories

Leadership and the theory behind what makes leaders “leaders” is a complex and intermingled topic. Transformational, leader-exchange, servant, and transactional theories are just a few of the proposed frameworks discussed in the literature to date [1]. These theories analyze facets such as traits, behaviors, followership, approaches, skills, and personality to try to quantify the mystique of individual and organizational effectiveness. In this project, we chose to adopt the framework of the skills approach model. With this approach, the focus is on the skills that leaders use rather than specific inherent qualities that an individual may possess. In this way, leadership becomes something that can be cultivated and developed [1].

Robert Katz is credited with developing the skills approach in his 1955 leadership article, “Skills of an Effective Administrator,” published in the *Harvard Business Review* [2]. In fall of 1974, that article was reprinted, augmented with additional observations from Katz over the intervening twenty-year period [3]. The skills approach emphasized that leaders engaged three key skill domains during their leadership practice: technical, human, and conceptual [2, 3]. Essentially, technical skill translated to expertise in concrete deliverables such as process, design, and methodology in a given area. Human skills were those skills related to both inter- and intra-personal skills. Finally, conceptual skills described the ability to think both holistically and contextually [2, 3]. Initially, it was hypothesized that lower leadership levels generally depended on technical skills and that as an individual leader ascended to higher levels, human and conceptual skills increased in importance. Katz later asserted that leaders at all organizational strata required some level of competence across skill sets. Additionally, realities such as organizational infrastructure could influence the need for technical skills at a more senior level than was previously recognized [3].

### Recent context

Over the decades, additional research has identified how the emphasis on skills may shift with organizational structure and standing. For instance, Mumford, Campion, and Morgenson found that the emergence of and need to engage with more strategic and business-related skills corresponded with organizational standing [4]. Similarly, Connelly et al. found that leaders' “knowledge and skills appear to contribute uniquely to leader achievement beyond what general cognitive ability and motivation contribute, perhaps because they enable leaders to construct viable and realistic solutions to continually changing problems or situations they encounter” [5]. Mumford et al. frame skills in the context of knowledge, problem-solving skills, social construction skills, and social judgment [6]. They also observed that the contextual use of knowledge and relevant mental models underpinned the successful deployment of abilities such as social skills [6]. Furthermore, more recently recognized skills such as forecasting, creative thinking, constraint analysis, and visioning among other cognitive-based skills warrant consideration in future leadership conversations and research [7].

Complex, multidimensional problems could be more likely to emerge with the expanded portfolio of senior health sciences library leadership positions. In today's library landscape, some leaders are being asked to assume roles beyond simply library director or head librarian, with titles such as dean, vice provost, or other highly visible and outward facing roles. These roles put librarians squarely in the position of needing to lead in an interprofessional context. Therefore, it would make sense that a skills-based approach to leadership in an interprofessional context also be considered from the lens of health sciences librarian professional development.

To identify core leadership skills across disciplines closely affiliated with academic health sciences librarianship, to map out the availability of literature in each of the four domains, and to identify any potential areas for further research in health sciences librarianship, we conducted a mapping review [8, 9] of the existing literature from the last five years around leadership in academic medicine, nursing, hospital administration, and health sciences librarianship.

## METHODS

We searched the literature published from January 2014 to February 2019 on leadership skills in each of four professional domains: (1) academic medicine, (2) academic nursing, (3) hospital administration, and (4) health sciences librarianship. We selected this five-year window in order to reflect the most current conversations related to leadership skills in these domains. Search terms and synonyms were used for concepts related to the keywords: leader*, medicine, medical librarianship, hospital administration/management, nursing, academia, and skills/competencies.

We searched MEDLINE/PubMed, Scopus, and Business Source Complete databases and included retrieved articles that discussed leadership skills or competencies written in English. Other discipline-specific databases, such as library literature databases, were not included, because we chose to search databases that were interdisciplinary in nature and included journals relevant to all four domains, including health sciences librarianship. Additionally, the availability of full-text was required for inclusion, with full-text being acquired post-screening. We then used text analysis and descriptive analysis to extract skills that were mentioned and identify trends that were in the literature.

**Figure 1:**
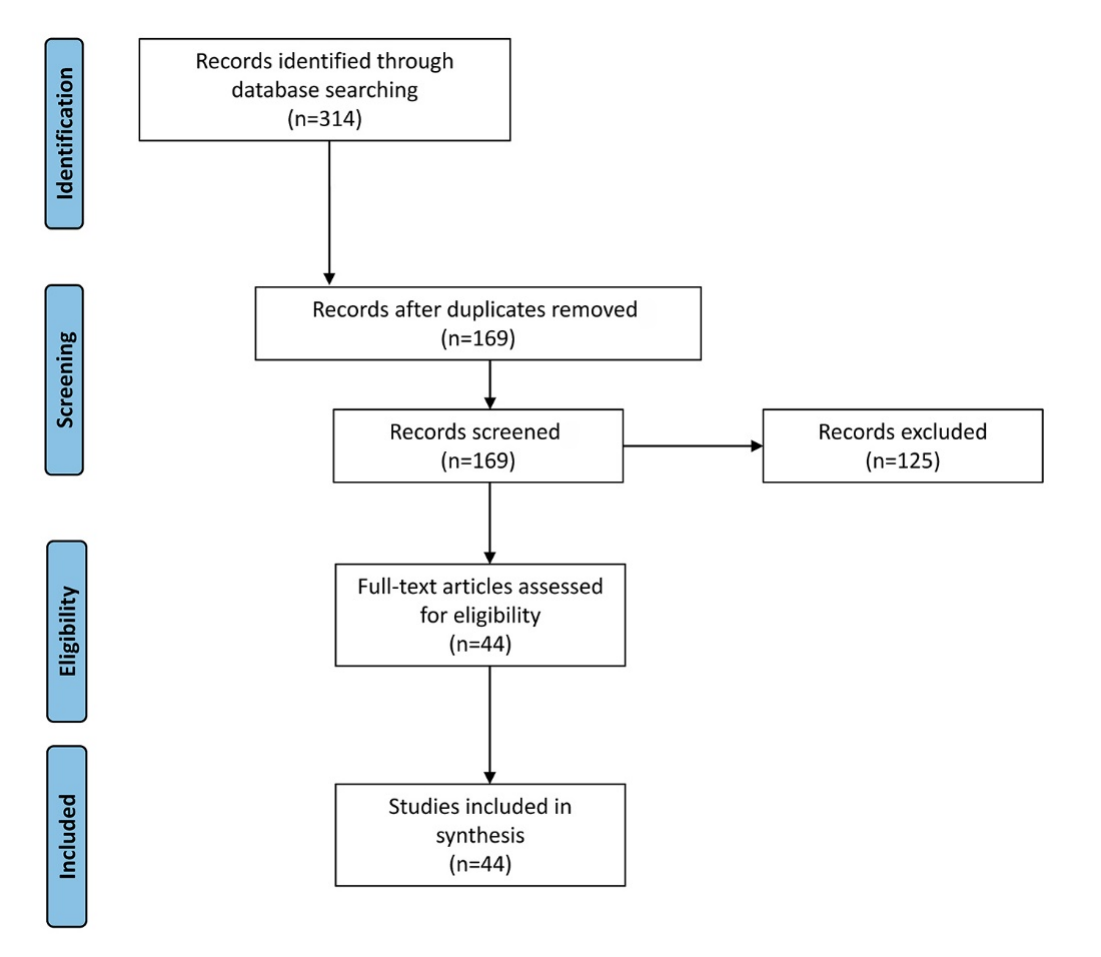
Flow diagram of article selection process

We retrieved 314 citations. After deduplication, we screened the remaining 169 unique citations based on our inclusion criteria (i.e., topic of leadership skills/competencies in 1 or more of the 4 professional domains, published in English, full-text available, published in last 5 years). As an example, an article discussing leadership challenges in an academic medical center but not discussing competencies or skills of effective leadership would have been excluded. After screening, we included 44 articles in the review (Figure 1). No citations were excluded due to lack of availability of full text.

Next, we retrieved and textually analyzed each of the included 44 articles (supplemental appendix) to identify and classify skills in each of the four identified domains. Text analysis was run using R [10] on all four professional domains as a whole, as well as on each domain individually. Word frequencies and patterns were examined using R [10], so that they could be ordered by domain and compared to existing frameworks [3, 11]. Upon recommendation by a data modeling and analysis consultant, we also examined individual documents in each domain for “term frequency/inverse document frequency” (tf-idf) to examine how important words in particular documents compared to the corpus of documents as a whole and to quantify what each document was about [12]. In other words, tf-idf weighs a term or keyword in any document and assigns importance to that term or keyword based on the number of times it appears [12]. In this way, the algorithm was able to judge the topics of a document by the words it contained.

Initially, for comparison's sake, we selected a published list of identified leadership skills [3, 11], then independently analyzed each of the word lists extracted in the specific domain and the leadership skills discussed therein to identify the most commonly discussed leadership skills in each of the four disciplines as they related to the published list. In cases of disagreement, we used a consensus method to discuss and resolve the variance between our opinions. Using this method, we achieved consensus on all documents in each domain. Subsequently, these skills were secondarily categorized using Katz's skills framework [3]. In comparing the text analysis to the existing frameworks, we did this first independently, capturing classification of items we agreed on. Any items that we did not agree on were discussed until consensus was reached.

## RESULTS

Of the 44 documents retrieved, most were in the academic medicine domain (n=20), followed by hospital administration (n=12), academic nursing (n=9), and health sciences librarianship (n=3) domains [13–56]. The 10 most frequently discussed skills in each domain, as represented by the Forbes Coaches Council list of skills [11] and as mapped to Katz's framework of 3 basic skills—technical, human, and conceptual [3]—are presented in Table 1.

**Table 1:** Forbes Coaches Council skills and Katz's skills mapped to each domain and from top-to-bottom in order of frequency (most frequent at top of table)

Academic nursing		Academic medicine		Hospital administration		Health sciences librarianship	
Forbes skills	Katz 3-skill approach	Forbes skills	Katz 3-skill approach	Forbes skills	Katz 3-skill approach	Forbes skills	Katz 3-skill approach
Change	Conceptual	Change	Conceptual	Learning	Technical	Vision	Conceptual
Communication	Human	Communication	Human	Change	Conceptual	Change	Conceptual
Vision	Conceptual	Learning	Technical	Communication	Human	Communication	Human
Learning	Technical	Vision	Conceptual	Vision	Conceptual	Learning (tie)	Technical
Individuality	Human	Individuality	Human	Individuality	Human	Individuality (tie)	Human
Respect (tie)	Human	Cultural Intelligence	Human	Respect	Human	Authenticity	Human
Flexibility (tie)	Technical	Respect	Human	Empathy	Human	Respect (tie)	Human
Cultural Intelligence (tie)	Human	Listening	Human	Flexibility	Technical	Empathy (tie)	Human
Agility (tie)	Technical	Empathy (tie)	Human	Listening	Human	Listening (tie)	Human
Humility (tie)	Human	Flexibility (tie)	Technical	Cultural Intelligence (tie)	Human	Cultural Intelligence (tie)	Human
		Authenticity (tie)	Human	Authenticity (tie)	Human		

In relation to the Forbes Coaches Council list of skills [11], the following skills were common to all four domains, although their ranking in each domain was generally different: change, communication, cultural intelligence, individuality, learning, respect, and vision. For example, “change” was the most frequently mentioned Forbes Coaches Council skill in academic nursing and academic medicine, while in hospital administration and health sciences librarianship, “change” was second, behind “learning” and “vision,” respectively.

**Figure 2:**
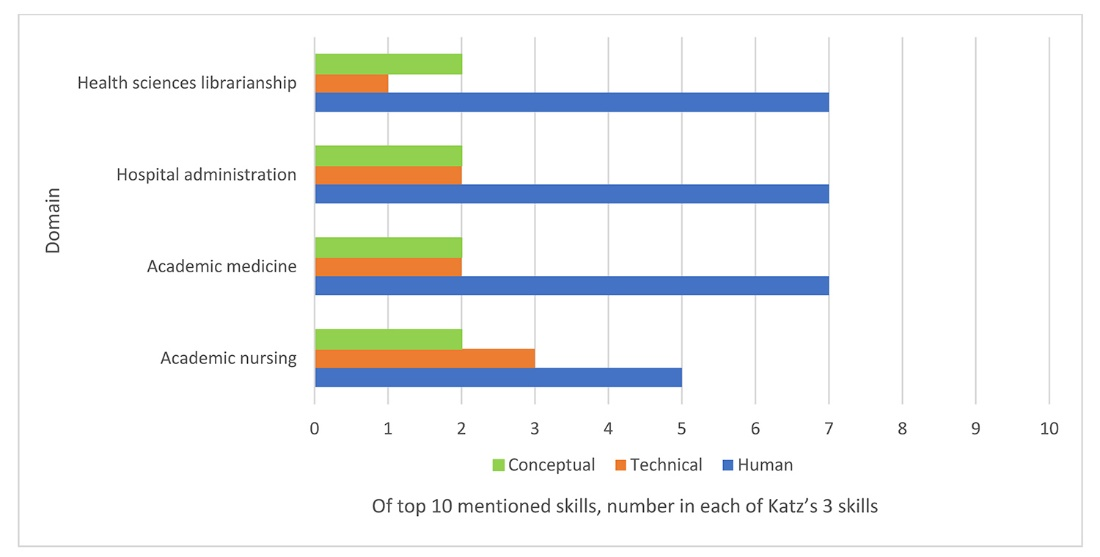
Leadership skills mapped to Katz three skills framework

When we mapped the top-10 leadership skills in each domain to Katz's 3 skills framework of human, technical, and conceptual skills (Figure 2), we noted that 70% of leadership skills mentioned in the health sciences librarianship leadership literature related to human skills, 20% to conceptual skills, and 10% to technical skills. Relative to “technical” and “conceptual” skills, “human” skills were mentioned most frequently across all 4 domains (academic nursing=50%, academic medicine=64%, hospital administration=64%, health sciences librarianship=70%). Academic nursing placed more emphasis on “technical” skills than the other domains, with health sciences librarianship placing the least emphasis on these skills. The 4 domains placed roughly equal emphasis on “conceptual” skills, with academic nursing and health sciences librarianship at 20% each, and academic medicine and hospital administration at 18% each.

The results of tf-idf analysis suggest that literature in the fields outside of health sciences librarianship describes leadership in more skills- and qualities-based terms than literature in health sciences librarianship, using terms such as “communication,” “empathetic,” “influence,” “integrity,” “self-awareness,” “skills,” “thoughtfully,” and “understand.” By contrast, articles in health sciences librarianship have tended to focus more on attributes common across existing librarians in leadership positions (e.g., “acumen,” “charismatic,” “director,” “passion”) rather than types of leadership skills and styles (e.g., transformational leadership) and how those skills work in a health care environment.

## DISCUSSION

Modern health sciences library leaders must more often lead—not only internally in the library setting, but also externally—as they are increasingly involved with connecting and collaborating with leaders across disciplines. Having the skills and vocabulary to lead and identify necessary leadership characteristics across fields is important to successfully navigate this landscape and enhance the impact of the library. By reviewing the current leadership literature in the fields that most often intersect with health sciences librarianship, librarians can better understand, learn, and enhance their own skills and vocabularies in these areas so as to better navigate the complex health care landscape.

Our literature search indicates that health sciences librarianship has recently published fewer articles on leadership than hospital administration, academic medicine, and academic nursing have. Only three articles from health sciences librarianship were retrieved out of the entire set of forty-four documents, showing that recent publications related to leadership skills and competencies in health sciences librarianship has lagged in comparison with the other three domains examined. This finding was both interesting and surprising and might suggest a gap in the professional literature. There could be several rationales for why this might be the case. For instance, health sciences librarians might more often read and draw on leadership literature published in other fields rather than literature specific to health sciences librarianship. An additional explanation could relate to differences in the population size of the four disciplines that were reviewed. For example, if there were fewer members in the health sciences librarianship profession than in the other professions, fewer publications in health sciences librarianship might be expected. Lastly, additional leadership skills and competencies literature for health sciences librarians might exist in the older professional literature for the field.

It was also noteworthy that the top five skills in each domain that mapped to the Forbes Coaches Council leadership skills [11]—“change,” “communication,” “individuality”, “learning,” and “vision”—were the same across domains, though they were in slightly different order for each domain. However, the next most frequently mentioned skills that mapped to the Forbes Coaches Council leadership skills [11] showed more variation across domains. For example, “flexibility” was not a top-ten skill mentioned in the health sciences librarianship articles but was in the top ten for each of the other domains. It was also interesting to note that across all four domains, skills in each discipline mapped most closely to Katz's human skills, which was to be expected because much of leadership depends on the ability to understand and motivate individuals and groups [2, 3].

On the whole, it is helpful to know that many leadership skills that are valued in the health sciences librarianship literature are similar to those that are valued in hospital administration, academic nursing, and academic medicine. We infer from this that the skills that are most often mentioned or valued in health sciences librarianship match closely to those in the domains that librarians most often interact with. Further research in the future examining reasons for the differences among the domains could additionally lead to interesting results and insights that explain some of these differences.

As Katz contended in his works [2, 3], framing leadership in terms of skills that can be taught, practiced, and learned, rather than as inherent qualities of or attributes about who leaders are, can help elevate leadership skills. The documents that we examined in this study suggest that in the health sciences librarianship profession, more discussion and training could be formed around leadership skills (e.g., technical, human, conceptual), similar to the ways in which leadership is described in academic medicine, academic nursing, and hospital administration.

As noted, across all four domains, human skills are relied upon heavily by those in leadership roles. [2, 3]. To work interprofessionally with leaders in academic nursing, academic medicine, and hospital administration, it is thus expected that health sciences library leaders may be able to closely align on human-centered skills, whereas differences in domain technical expertise may be more difficult to span. For future training considerations, conceptual-related skills may be an interesting area for health sciences librarian leaders to practice and expand upon their existing skill sets. According to Katz, conceptual skills become increasingly important in higher executive positions, and it is at this level that health sciences librarian leaders can most effectively leverage integrations with the other domains by articulating librarians' systemic roles and impact in the greater organization. Being able to ascertain and compellingly articulate concepts related to how the library integrates into larger organizational policy decisions and administrative processes through a shared leadership language could be highly useful skills for health sciences library leaders to more formally learn and practice.

Our review did have some limitations. While we systematically searched the literature on this topic, the project was designed as a mapping review [8, 9] and not as a systematic review, and, thus, there might be additional relevant literature that we did not retrieve. For instance, information science–specific databases and literature prior to 2014 were not included. Literature in languages other than English was also excluded, as we did not have cross-language textual analytic capabilities available to us. Additionally, the initial skills list that we used for comparison was limited in scope. Other skills and competencies in the domains that were not discussed in this article might be relevant and warrant further consideration. However, the initial findings of this project may provide a valuable opening stage toward more focused evidence reviews or systematic reviews on this topic, as well as opportunities for additional research and publication on leadership in health sciences librarianship.

Health sciences librarian leaders work every day with colleagues in academic medicine, academic nursing, hospital administration, and other health professions, and we librarians would be well served to expand our leadership vocabulary and skills to better understand, reflect, and incorporate skills and attributes that are valued interprofessionally. By identifying and framing ourselves through this shared vocabulary, we may be better positioned in the future to increase our advocacy and articulate the value of the library by leveraging this universal language to our advantage. Addressing the current gap and expanding the literature in the area of health sciences library leadership is a natural next step. Additionally, examining and developing training specifically informed by the leadership literature across health disciplines might position us more strategically to better align with and lead alongside our health professions colleagues.

## SUPPLEMENTAL FILE

**Appendix:** EndNote library
